# Glycidyl Methacrylate-Based Copolymers as Healing Agents of Waterborne Polyurethanes

**DOI:** 10.3390/ijms23158118

**Published:** 2022-07-23

**Authors:** Ioanna Tzoumani, Amaia Soto Beobide, Zacharoula Iatridi, George A. Voyiatzis, Georgios Bokias, Joannis K. Kallitsis

**Affiliations:** 1 Department of Chemistry, University of Patras, GR-26504 Patras, Greece; tzoumani@upnet.gr (I.T.); asoto@iceht.forth.gr (A.S.B.); bokias@upatras.gr (G.B.); kallitsi@upatras.gr (J.K.K.); 2 FORTH/ICE-HT, Stadiou Street, P.O. Box 1414, GR-26504 Patras, Greece; gvog@iceht.forth.gr

**Keywords:** waterborne polyurethane, glycidyl methacrylate, n-butyl acrylate, poly(ethylene glycol) methyl ether methacrylate, free radical polymerization, self-healing, temperature trigger, water trigger

## Abstract

Self-healing materials and self-healing mechanisms are two topics that have attracted huge scientific interest in recent decades. Macromolecular chemistry can provide appropriately tailored functional polymers with desired healing properties. Herein, we report the incorporation of glycidyl methacrylate-based (GMA) copolymers in waterborne polyurethanes (WPUs) and the study of their potential healing ability. Two types of copolymers were synthesized, namely the hydrophobic P(BA-co-GMAy) copolymers of GMA with n-butyl acrylate (BA) and the amphiphilic copolymers P(PEGMA-co-GMAy) of GMA with a poly(ethylene glycol) methyl ether methacrylate (PEGMA) macromonomer. We demonstrate that the blending of these types of copolymers with two WPUs leads to homogenous composites. While the addition of P(BA-co-GMAy) in the WPUs leads to amorphous materials, the addition of P(PEGMA-co-GMAy) copolymers leads to hybrid composite systems varying from amorphous to semi-crystalline, depending on copolymer or blend composition. The healing efficiency of these copolymers was explored upon application of two external triggers (addition of water or heating). Promising healing results were exhibited by the final composites when water was used as a healing trigger.

## 1. Introduction

Self-healing materials are materials that can partially or completely regain their original properties after physical damage [1,2,3,4,5,6]. Two main self-healing mechanisms have been proposed to ensure the durability of materials or coatings: (a) Extrinsic self-healing wherein a healing agent needs to be introduced in the material during the synthetic/production procedure. In this case, the healing agent is usually encapsulated in reservoirs like microcapsules, microfibers or vascular networks which are dispersed in the matrix (usually polymeric). After a damage, usually a crack, these encapsulants are ruptured, releasing the healing agent in the matrix [7,8]. (b) Intrinsic self-healing in which the materials can heal a damage themselves, without the addition of an external healing agent. Herein, the healing process mechanism is governed by chemical or physical interactions [9,10,11,12]. Thus, chemical interactions like Diels-Alder reactions [13], imine bonds [14], disulfide bonds [15,16,17], boronic ester crosslinks [18], as well as physical interactions like hydrogen bonding [19,20,21], ionic bonds [22], hydrophobic or electrostatic interactions and host-guest interactions [23], can provide healing. Self-healing materials can also be categorized as autonomous or non-autonomous materials [6,7,24]. In autonomous systems, healing happens without external triggers while in non-autonomous systems, external trigger (usually heat, light etc.) is necessary to initiate the healing process. 

Polyurethanes (PUs) and especially waterborne polyurethanes (WPUs) are high performance elastomers with interesting chemical structure and environmentally friendly character. However, they are quite vulnerable to damage (by mechanical, chemical, thermal, or other reasons), leading to undesired scratches or notches that eventually affect the products lifetime. This susceptibility makes the production of PUs with improved functions, like self-healing, flexibility, elasticity, abrasion resistance, an emerging demand. Such characteristics can be yielded in the PUs and WPUs by adding appropriate functional groups, usually via chemical reactions [13]. 

Recent trends on healing of various coatings are based on blending. This way, by simply mixing, for example, WPU dispersions with water-soluble “fillers” (polymers, organic or inorganic particles, etc.), environmentally friendly composites with modified mechanical and other properties (abrasion resistance, healing, processability) can be easily manufactured, without requiring complex synthesis and chemical modification processes. Some current studies report the fabrication of self-healable nanocomposites based on blends of PUs and organic or inorganic nano- or micro-particles [25,26,27,28]. Other studies have shown that the incorporation of adequately functional polymers or other compounds (for instance, poly(dopamine methacrylamide) [29], polycaprolactone [30], tannic acid [31]) as the dispersed phase in polyurethane matrices leads to self-healable composites. In such systems, self-healing is based on hydrogen bonding, van der Waals forces, hydrophobic interactions, thermo-induced healing, etc. 

Epoxy-functional polymers are recognized as versatile polymeric materials with a wide range of industrial applications. Poly(glycidyl methacrylate) (PGMA) is one of the most interesting functional macromolecules with side epoxy groups. Glycidyl methacrylate (GMA) is a low cost, non-toxic and highly reactive hydrophobic monomer, which can be easily copolymerized with a variety of monomers, leading to advanced materials. Moreover, GMA can serve as a precursor of multifunctional polymers, since its pendant epoxy group can react with nucleophiles (e.g., amines, carboxylic acids, thiols, etc.), creating new polymeric architectures with additional functionalities or responsive abilities [32]. Copolymers of GMA, like poly(ethylene-co-glycidyl methacrylate), have been used as healing thermoplastic modifiers in epoxy/amine resin [33], while the GMA monomer has been exploited as a healing agent in GMA-loaded microcapsules in epoxy or poly(methyl methacrylate) (PMMA) resins [34,35]. On the other hand, it has been shown that hydrophobic copolymers made from common monomers, like MMA and n-butyl acrylate (BA), can repair a damage either without external intervention via weak attractive van der Waals forces [36] or in the presence of water (water-accelerated self-healing) [37].

Among others, GMA has been combined with the soft and hydrophobic n-butyl acrylate (BA) monomer or poly(ethylene glycol) methyl ether methacrylate (PEGMA) macromonomers leading to materials with low glass transition temperature. Copolymers of GMA and BA have been utilized as latexes or for the reinforcement of epoxy resins [38,39,40,41], while copolymers of GMA and PEGMA can find use in bioapplications (wound dressings [42], drug delivery systems [43], antibacterial nanocarriers [44], enzyme-polymer conjugates [45], as solid [46,47] or gel [48] polymer electrolyte membranes or as coatings for the preparation of modified membranes with antifouling [49] or heavy metals and dyes separation [50] properties. 

Taking advantage of the reactivity of GMA-based copolymers towards acrylic acid units or amine groups, the preparation of coatings for potential antimicrobial or antifouling applications is elaborated in our laboratory during the last years [51,52,53], while through the combination with collagen, the preparation of hydrogels as potential biofertilizers has been recently explored [54]. 

As has been previously reported, the introduction of ethylene glycol groups contributes to effective water mediated [55,56] or thermally mendable [57,58] self-healing behavior of films or coatings, mainly due to the increasing chain mobility, offered by the triggers. For example, upon exposure to water or moisture, polyethylene glycol provides chain fluidity, filling the damage (e.g., cracks or micrometer-sized cuts), while it can potentially be involved in healing mechanisms like hydrogen-bond formation with supplementary moieties [55,56,59]. Based on these concepts, the design principle in our study was to synthesize functional copolymers that combine reactive groups with units that can be activated by triggers like water or temperature changes. Thus, in the present work, a series of random GMA-based copolymers with the hydrophobic monomer BA or a hydrophilic PEGMA macromonomer were synthesized. Such polymeric structures combine the functionality of GMA with additional properties, like the control of hydrophilic/hydrophobic balance and modification of the thermal properties. These copolymers were used as additives in waterborne polyurethane resins and their healing potential upon application of external triggers, like water or heat, was explored. Both WPU/P(BA-co-GMA) and WPU/P(PEGMA-co-GMA) composite systems exhibited promising water-mediated healing efficiency, suggesting their future use as potential healable coatings.

## 2. Results

### 2.1. Copolymer Characterization

**Scheme 1:**
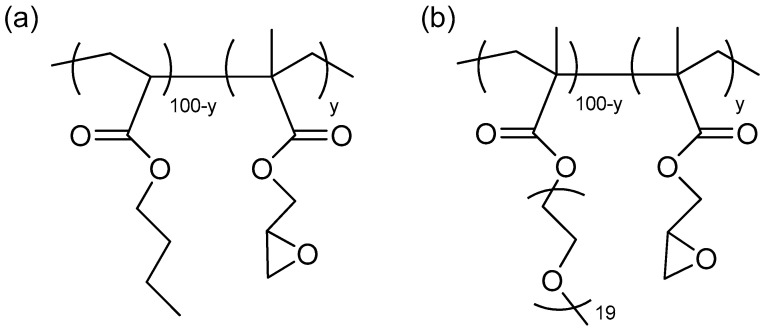
Structure of the (**a**) P(BA-co-GMAy) and (**b**) P(PEGMA-co-GMAy) copolymers.

Two functional copolymer structures have been synthesized and tested as potential healing additives in WPUs. In the first case, GMA units were combined with hydrophobic BA units to prepare water-insoluble P(BA-co-GMAy) copolymers, while in the second case, GMA was copolymerized with a hydrophilic PEGMA macromonomer (Mn 950 g/mol) to produce water-soluble amphiphilic P(PEGMA-co-GMAy) copolymers. The structure of these two copolymer families, where y is the molar feed content in GMA units, is presented in Scheme 1. The copolymers were synthesized through free radical copolymerization and characterized by several techniques like ^1^H-NMR, SEC, ATR-FTIR and DSC. 

**Figure 1:**
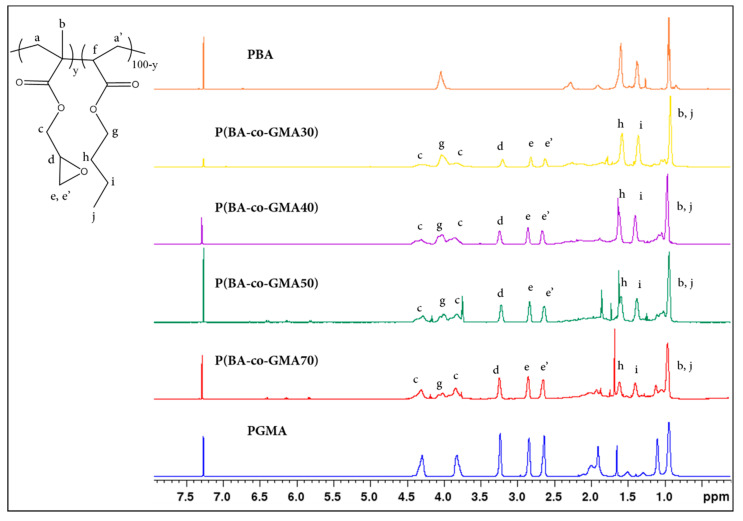
^1^H-NMR spectra of the P(BA-*co*-GMAy) copolymers in CDCl_3_. The spectra of the homopolymers PGMA and poly(n-butyl acrylate), PBA, are also presented.

The chemical composition of the synthesized copolymers was determined through ^1^H-NMR spectroscopy. Figure 1 shows the ^1^H-NMR spectra in CDCl_3_ of the P(BA-co-GMAy) copolymers with y = 30, 40, 50 and 70. The presence of GMA in the final copolymers is confirmed by the characteristic peaks at 3.85 and 4.31 ppm corresponding to the methylene protons (c) of GMA. The peak at 3.26 ppm is attributed to the methyl proton (d) and the peaks at 2.7 and 2.9 ppm correspond to the methylene protons (e, e’) of the epoxy ring. The methyl protons (j, b) of BA and GMA are observed at 0.79–1.21 ppm. The peak at 1.39 ppm corresponds to methylene protons (i), while the peak at 1.61 ppm corresponds to methylene protons (h). Finally, the peak at 4.03 ppm is attributed to the methylene protons (g) of BA. 

The molar ratio of GMA in the P(BA-*co*-GMAy) copolymers was calculated from the integrals of the signals at 3.26 ppm (d, GMA unit) and 1.39 ppm (i, BA unit). As seen in Table 1, the actual composition of the copolymers is in a rather good agreement with the feed composition. The number-average molar mass (*M*_n_), the weight-average molar mass (*M*_w_) and the polydispersity index (PDI), as obtained from SEC, are also given in Table 1. As seen, copolymers with reasonably high molar masses are obtained (*M*_w_ is in the range ~2–5 × 10^4^ g/mol), while PDI values are typical for free radical polymerzation products. 

**Figure 2:**
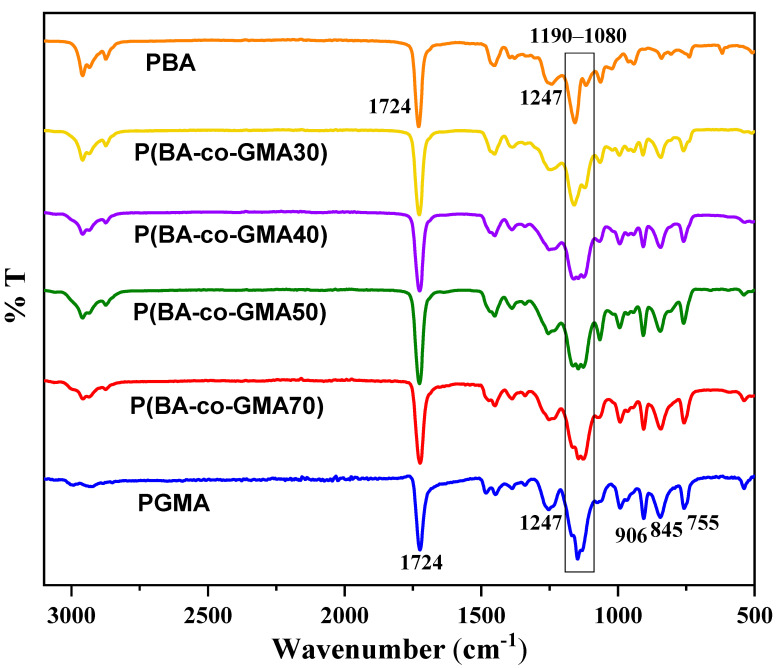
ATR-FTIR spectra of the P(BA-co-GMAy) copolymers. The spectra of the homopolymers PBA and PGMA are also presented.

The synthesized copolymers were further examined by ATR-FTIR spectroscopy, and the spectra are shown in Figure 2. For comparison, the spectra of PBA and PGMA homopolymers are also shown. The characteristic stretching vibration of the ester group is observed at 1724 cm^−1^, which is related to the carbonyl C=O group of BA and GMA units. Moreover, the peaks at 1247 cm^−1^ and in the region 1080–1190 cm^−1^ correspond to the stretching vibration of the C-O bond of both BA and GMA. It is clear, especially in the region of 1080–1190 cm^−1^, that the peaks of the copolymers are broader, as a result of the superposition of the corresponding peaks of the PBA and PGMA homopolymers. The presence of a GMA unit in the copolymers P(BA-co-GMAy) is further verified by the characteristic bands at 845 and 906 cm^−1^, attributed to the vibrations of the epoxy ring. The peak, also, at 755 cm^−1^ corresponds to the bending vibration of the CH group of the epoxy ring. 

**Figure 3:**
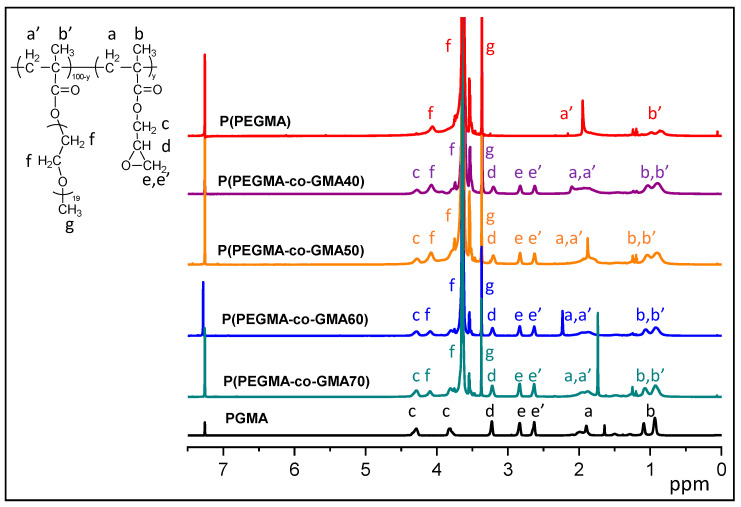
^1^H-NMR spectra of the P(PEGMA-*co*-GMAy) copolymers in CDCl_3_. The spectra of the homopolymers P(PEGMA) and PGMA are also presented.

The characterization of the P(PEGMA-co-GMAy) copolymers by ^1^H-NMR is shown in Figure 3. The spectra of the homopolymers PGMA and poly(poly(ethylene glycol) methyl ether methacrylate), P(PEGMA), are also presented. The signals at 0.8–1.2 ppm and 1.2–2 ppm are attributed to the protons of the methylene groups –CH_3_ (b, b’) and the –CH_2_– (a, a’) groups of the main chain, respectively. The protons of the –CH_2_– group of the epoxide ring of GMA (e) can be detected at 2.7 and 2.9 ppm, while the proton of the –CH– group (d) is seen at 3.26 ppm. Moreover, the signal at 4.31 ppm corresponds to the methylene protons –O-CH_2_– (c) adjacent to the epoxy groups of GMA. The signals at 3.38 ppm and 3.6 ppm are assigned to the protons of the methylene group –CH_3_ of PEGMA (g) and the protons of the CH_2_-CH_2_-O (f) groups of PEGMA units, respectively. The monomer molar ratio of the polymers was determined comparing the signals at 3.38 ppm (PEGMA unit) and at 3.26 ppm (GMA unit). The calculations for both families of copolymers are presented in detail in Figure S1, Tables S1 and S2.

The characterization results from ^1^H NMR along with the SEC characterization (*M*_n_, *M*_w_, PDI) are presented in Table 2. As seen, the resulting composition of the copolymers is in a rather good agreement with the feed composition. For this series of copolymers, products with quite high molar masses are obtained (*M*_w_ is in the range ~1–5 × 10^5^ g/mol). These high values are understandable, since the copolymers bear high contents of PEGMA units with a molar mass around 1000 g/mol. 

**Figure 4:**
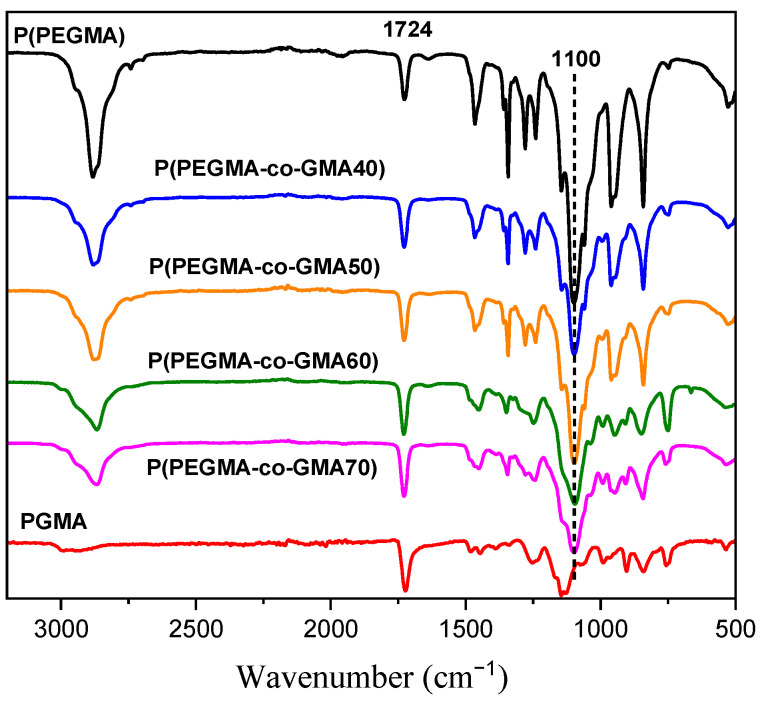
ATR−FTIR spectra of the P(PEGMA-*co*-GMAy) copolymers. The spectra of P(PEGMA) and PGMA homopolymers are also presented.

The ATR-FTIR characterization of the P(PEGMA-co-GMAy) copolymers is presented in Figure 4. The characteristic peak at 1724 cm^−1^ corresponds to the stretching vibrations of the ester groups (C=O) of both GMA and PEGMA and was utilized for the normalization of the spectra. The peak at 1100 cm^−1^ is attributed to the characteristic stretching vibration of the ether bonds (C-O-C) of PEGMA. It can be observed that the ratio of the intensities of these two peaks decreases with increasing the GMA content, implying the existence of both PEGMA and GMA units in the copolymers, according to the feed composition. The band around 1460 cm^−1^ is attributed to C-H stretching and bending modes of methylene groups of PEGMA and GMA. The peaks at 755 and 906 cm^−1^ are attributed to the epoxide groups of GMA. These peaks are not detected in the spectrum of P(PEGMA) while they are present in the spectra of the P(PEGMA-co-GMAy) copolymers. The same counts for the peak of the twisting mode of the methylene groups of the side chain of PEGMA at ~1300 cm^−1^; it is not detected in the spectrum of PGMA, while it is present in the spectra of the P(PEGMA-co-GMAy) copolymers.

**Figure 5:**
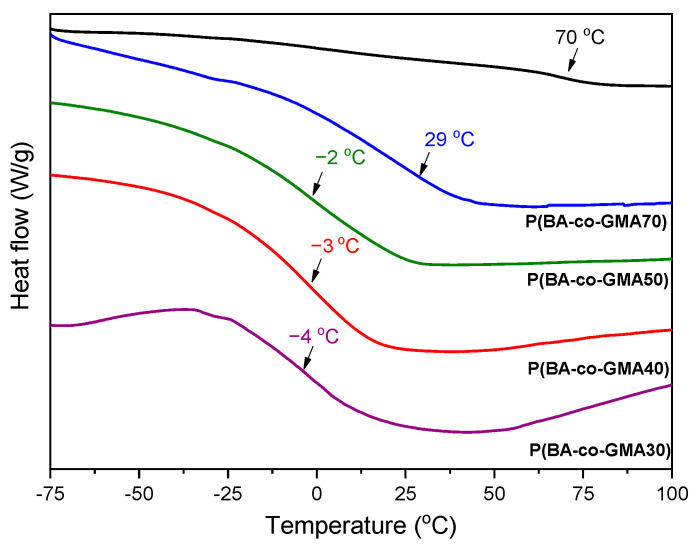
DSC curves of first heating cycle for the P(BA-co-GMAy) copolymers.

The thermal behavior of the resulting P(BA-co-GMAy) copolymers is shown in Figure 5. All products in this copolymer series are amorphous and just the glass transition temperature, Tg, is detected. The Tg of PBA is known to be very low, −53 °C [60], while the Tg of PGMA is found at 70 °C, close to the Tg values reported in literature [61,62]. It is seen that the Tg of the copolymers takes values between the Tg of the two homopolymers. As a general trend (Figure S2), the Tg values of the copolymers increase regularly with increasing the actual mole fraction of GMA, indicating a good compatibility between the two structural units. Interestingly, when the actual composition of the copolymers is close to molar stoichiometry (copolymers with y = 40 and 50, containing 46 and 55 %mol GMA), the Tg value is around −3 °C/−2 °C, namely about 20 °C lower than the expected one (following the Tg trend shown in Figure S2). Though this observation should be further verified and systematically studied, it might be an indication that, when the copolymer composition is close to molar stoichiometry, the macromolecular chains adapt conformations allowing higher free volumes in bulk. 

**Figure 6:**
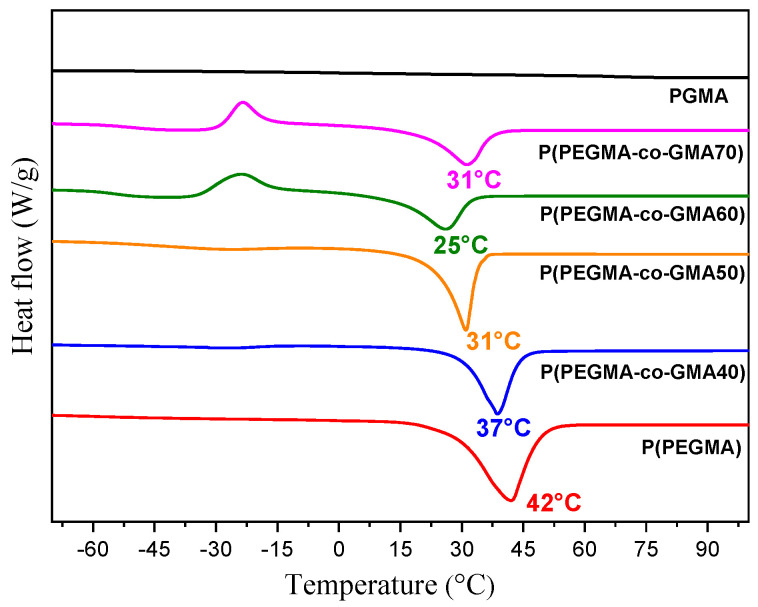
DSC curves of first heating cycle for the P(PEGMA-co-GMAy) copolymers. The spectra of the homopolymer P(PEGMA) and PGMA are also presented.

The DSC thermograms for P(PEGMA-co-GMAy) copolymers, along with the thermograms of PGMA and P(PEGMA) homopolymers as reference are depicted in Figure 6. The thermal characteristic values obtained by DSC are tabulated in Table 3. As a consequence of the very high PEGMA weight content of these copolymer series, the thermal behavior seems to be governed by the presence of PEGMA. Thus, all P(PEGMA-co-GMAy) copolymers exhibited a single Tg between −43 and −55 °C (Figure S3), namely somewhat higher than the Tg of P(PEGMA), −70 °C. Similar findings have been reported by Borodinov et al. [63]. As can be seen, the Tg values of the copolymers are closer to that of pure P(PEGMA), because of the high weight fraction of the PEGMA macromonomer segments. In addition, all P(PEGMA-co-GMAy) copolymers exhibit crystallinity, attributed to the side PEGMA chains. The melting temperature, Tm, values of the copolymers are lower than the Tm of pure P(PEGMA), indicating that the introduction of GMA units destroys the packing regularity of PEGMA chains, leading to the melting point decrease. It is evident that the Tm values as well as the enthalpies of melting are strongly influenced by the composition of the copolymer. For PEGMA content 30 and 40 mol%, the copolymers exhibit an obvious crystallization peak and a melting peak during heating. These copolymers are considered as semicrystalline with the possibility of thermal-induced crystallization at a crystallization temperature, Tc. For PEGMA compositions 50 and 60 mol%, the resulted copolymers are crystalline exhibiting only a melting temperature but not crystallization temperature. The degree of crystallinity, X_C_%, of the copolymers is calculated from the thermographs using the equation:

(1) $$X_{C}\%=\frac{{\Delta H}_{m}-{\Delta H}_{c\;}}{{\Delta H}_{m\;}^{ο}\;}*100$$

where ΔH_m_ is the enthalpy of melting and ΔH_c_ is the enthalpy of crystallization, both determined by DSC. ΔH_m_ is the enthalpy of melting of a completely crystalline material. In our case we used the value ΔH_m_ = 197 J/g, corresponding to poly(ethylene glycol), considered as the more similar structure to PEGMA [64,65]. From Equation (1), the crystallinity of the whole copolymer is determined. Since the only crystallizable unit is PEGMA, %X’_C_ reports the “corrected” crystallinity, when only the actual content of PEGMA units in the copolymers is considered. As a general tendency, the percentage of crystallinity of the copolymers decreases with the increase of the GMA content due to irregularities in PEGMA molecular packing. In combination with the very low Tg, this effect is considered as a main advantage on the use of these copolymers as self-healing agents, in case they are embedded in a coating matrix, since they can easily be mobilized when a scratch is formed. 

### 2.2. Investigation of WPU/Copolymer Films

For the preparation of the WPU/copolymer composites, WPU dispersions were mixed with solutions of the as synthesized copolymers, spread on glass petri dishes or Teflon sheet, and left to stand at ambient temperature until full evaporation of solvent and formation of films. Two laboratory-made WPUs, coded WPU1 and WPU2, were tested. Two mixing contents, namely 10% *w*/*w* and 20% *w*/*w* on dry basis, were studied. The nomenclature used for these composites is WPUi/xcopolymer, where i = 1 or 2 and x is the weight content, 10 or 20 (in % *w*/*w*). The aim was to use as little additive as possible in the WPUs, in order to minimize the influence in the final properties (e.g., transparency, homogeneity, mechanical properties, etc.) of the polyurethane film. The formed films seem to be homogeneous, quite transparent and non-brittle.

**Figure 7:**
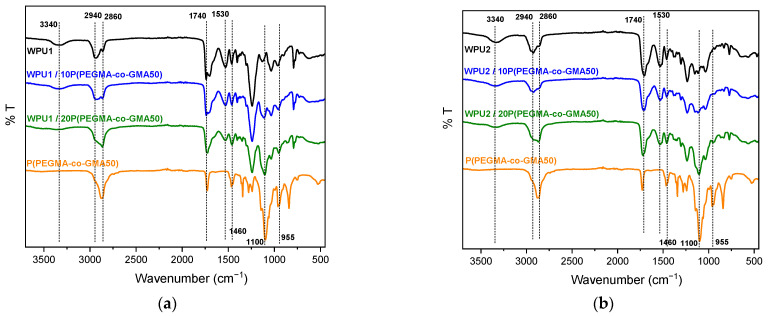
ATR-FTIR spectra of (**a**) WPU1/P(PEGMA-co-GMA50) and (**b**) WPU2/P(PEGMA-co-GMA50) films containing P(PEGMA-co-GMA50) in two weight ratios (10 and 20% *w*/*w*). The spectra of P(PEGMA-co-GMA50) copolymer and the films of pure WPU1 and WPU2 are also presented.

ATR-FTIR and DSC were employed to verify the chemical composition and explore the thermal properties of the WPU/GMA-based copolymer composites. In Figure 7, the ATR-FTIR spectra of WPU/P(PEGMA-co-GMA50) films are shown, as example. For reasons of comparison, the ATR-FTIR spectra of films consisting only of the corresponding polyurethane film and P(PEGMA-co-GMA50) are also given. In the spectra of pure WPUs, characteristic absorption peaks of polyurethanes can be detected [66,67] Indeed, the broad absorption band at 3340 cm^−1^ corresponds to the stretching vibration of the N-H groups (Amide I) of polyurethane. The absorption peaks located at ~2940 and 2860 cm^−1^ are attributed to the asymmetric and symmetric stretching CH_2_ vibrations of alkanes. The peak of non-hydrogen bonded carbonyl urethane group C=O is seen at 1740 cm^−1^. The absorption band at 1530 cm^−1^ is attributed to the bending vibration of N-H of urethane (Amide II), while the peaks in the range of 1100–1350 cm^−1^ indicate stretching vibration of O-C=O and C-N groups of urethanes. In the spectra of the WPU/P(PEGMA-co-GMA50) composites, the abovementioned characteristic peaks of the WPUs, as well as of the P(PEGMA-co-GMA50) copolymer, can be identified. It can be observed that as the copolymer P(PEGMA-co-GMA50) content increases in the WPU/P(PEGMA-co-GMA50) composites, some absorption bands, which are characteristic for the P(PEGMA-co-GMA50) copolymer (1460 cm^−1^, 955 cm^−1^ and especially the peak at 1100 cm^−1^), are enhanced in the ATR-FTIR spectra of WPU1/P(PEGMA-co-GMA50) and WPU2/P(PEGMA-co-GMA50). Therefore, it can be confirmed that the copolymer P(PEGMA-co-GMA50) has been successfully incorporated into the polyurethane films. 

**Figure 8:**
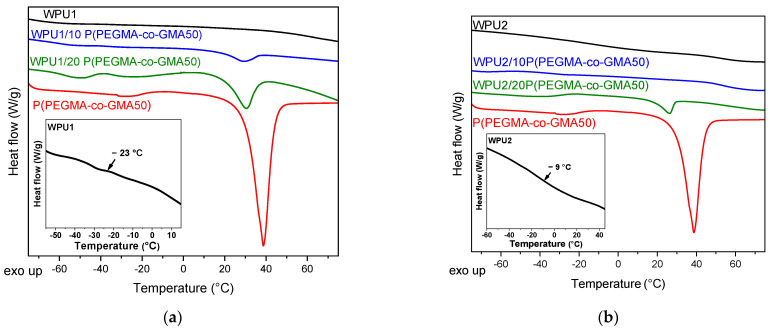
DSC of the (**a**) WPU1/P(PEGMA-co-GMA50) and (**b**) WPU2/P(PEGMA-co-GMA50) films containing P(PEGMA-co-GMA50) in two different weight ratios (10 and 20 %wt). P(PEGMA-co-GMA50) copolymer and the films of pure WPU1 and WPU2 are also presented.

The thermal behavior of the WPU/copolymer films was studied by DSC. A summary of all DSC results for P(PEGMA-co-GMA) and P(BA-co-GMA) copolymers, as well as of WPUs and WPU/copolymer films are presented in Table 4. In Figure 8, the thermograms of pure WPU1 and WPU2, the copolymer P(PEGMA-co-GMA50) and the composite films of WPU1 or WPU2 with P(PEGMA-co-GMA50), at 10 and 20% *w*/*w* polymer weight contents, are shown. The blends exhibit a characteristic behavior of both WPUs and the P(PEGMA-co-GMA50) polymer. As shown in Figure 8a, while the Tg of WPU1 is −23 °C, after the incorporation of 10% *w*/*w* or 20% *w*/*w* P(PEGMA-co-GMA50) polymer content, the Tg of the composites decreases to −37 °C and −34 °C, respectively (Table 4). Likewise, in the case of WPU2 blends (Figure 8b), the WPU2/10P(PEGMA-co-GMA50) and WPU2/20P(PEGMA-co-GMA50) composites exhibited lower Tg values (−28 °C and −32 °C, respectively), when the Tg of WPU2 is −9 °C (Table 4). These Tg values of all the WPU1/P(PEGMA-co-GMA50) and WPU2/P(PEGMA-co-GMA50) composites, suggest that the formed composite films are characterized by good compatibility of their individual components. 

The Tg of the WPU2/10P(PEGMA-co-GMA70) was found to be −3 °C, a value close to the value found for pure WPU2 and much higher than that of the corresponding WPU2/10P(PEGMA-co-GMA50) composite (Tg = −28 °C). This difference probably indicates a poor compatibility of the two ingredients (WPU2 and P(PEGMA-co-GMA70)) of this composite system.

The WPU1/10P(PEGMA-co-GMA50), WPU1/20P(PEGMA-co-GMA50) and WPU2/20P(PEGMA-co-GMA50) films presented Tm at 28 °C, 30.5 °C and 26.5 °C, respectively, showing a significant melting point depression, as compared to the Tm value (37 °C) of the pure copolymer. For these samples, the crystallinity %X″c was determined after taking into account the actual copolymer composition, corresponding to %X^’^c, and the film composition. A strong reduction of the crystallinity of PEGMA segments is observed after mixing, enabling thus the amorphous copolymer phase to interact with the PU resin, as supported also by the Tg values.

### 2.3. Self-Healing Tests

For the study of the healing capability of the WPU/polymer composite films, the films were scratched by a razor blade and the created scratches were monitored by an optical microscope. 

As an initial study, we proceeded in the preparation of a blend containing WPU1 and the pure P(PEGMA) homopolymer (polymer mass content on dry basis in the blend: x = 10% *w*/*w*), namely WPU1/10P(PEGMA). This composite gave a film that did not exhibit any scratch healing efficiency, neither by adding droplets of water on the scratch, nor by heating the scratched film. This behavior possibly indicates the importance of the presence of the GMA content in the copolymers, to also contribute to the healing process. 

**Figure 9:**
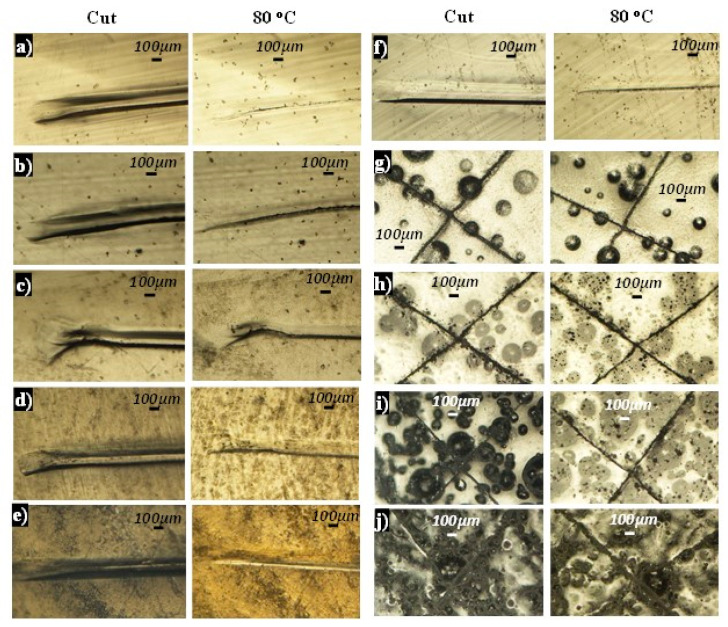
Optical microscopy images of scratch on (**a**) neat WPU1, (**b**) WPU1/10P(PEGMA-co-GMA40), (**c**) WPU1/20P(PEGMA-co-GMA40), (**d**) WPU1/10P(PEGMA-co-GMA50), (**e**) WPU1/20P(PEGMA-co-GMA50), (**f**) WPU1/10P(PEGMA-co-GMA70), (**g**) WPU1/10P(BA-co-GMA30), (**h**) WPU1/10P(BA-co-GMA40), (**i**) WPU1/10P(BA-co-GMA50) and (**j**) WPU1/10P(BA-co-GMA70) films, before (left photos) and after (right photos) heating at 80 °C.

Figure 9 shows the self-healing process of the WPU1/P(PEGMA-co-GMA) composite films, after placing the cut films in an oven at 80 °C for 12 h. As seen in Figure 9a, pure WPU1 exhibited almost full crack recovery. The Tg of WPU1 from DSC (Table 4) is as low as −23 °C. Thus, heating at 80 °C can cause heat-induced mobility and molecular diffusion of the soft segments of WPU1 across the interface, that leads to the formation of chain entanglements between the scratch surfaces, resulting eventually to its recovery. As far as the WPU1/polymer composites are concerned, the incorporation of the polymers into WPU1 resulted in a decrease of this self-healing behavior of WPU1. The cracks on the WPU1/P(PEGMA-co-GMA) films (Figure 9b–f) healed at a large extent, while the healing of the WPU1/P(BA-co-GMA) films (Figure 9g–j) was insignificant. By these findings it can be assumed that the nature of the WPUs and their thermal characteristics are the decisive factors ruling the behavior of the final composites.

**Figure 10:**
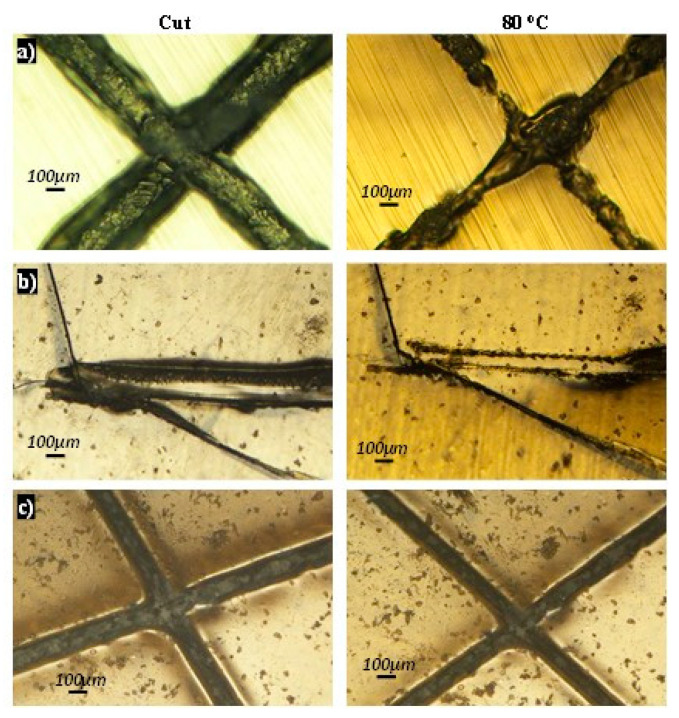
Optical microscopy images of scratches on (**a**) pure WPU2, (**b**) WPU2/10P(PEGMA-co-GMA50) and (**c**) WPU2/20P(PEGMA-co-GMA50) films, before (left photos) and after (right photos) heating the films at 80 °C.

In Figure 10 the healing progress of the WPU2/P(PEGMA-co-GMA) composite films is presented, after placing the films in an oven at 80 °C. Pure WPU2 exhibited a slight decrease of the cross scratch after exposure at 80 °C (Figure 10a), in contrast to the full recovery of the cut in the pure WPU1 film, as already shown in Figure 9a. The incorporation of the P(PEGMA-co-GMA50) copolymer in WPU2 (Figure 10b,c) did not ameliorate the healing behavior of the composite films. 

The heat induced self-healing potential of the WPU2/P(BA-co-GMAy) composite films was also explored. For this study, the WPU2/P(BA-co-GMAy) films were cross scratched and put in the oven at 80 °C, for 12 h. In Figure S5, no satisfying healing of the scratch is observed for all the studied films. It is noteworthy that the WPU2/P(BA-co-GMAy) composites with GMA content y = 30, 40 and 50% moles, exhibit a trend to repair the cross scratch upon heating, unlike the WPU2/P(BA-co-GMA70) composites, with a GMA content y = 70% moles, that displays zero healing capability. 

**Figure 11:**
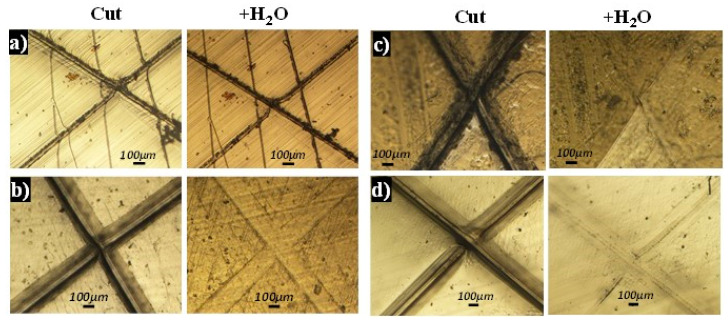
Optical microscopy images of cross scratch on (**a**) neat WPU1, (**b**) WPU1/10P(PEGMA-co-GMA50), (**c**) WPU1/20P(PEGMA-co-GMA50) and (**d**) WPU1/10P(PEGMA-co-GMA70), before (left photos) and after (right photos) addition of water on the cut.

Figure 11 shows the self-healing process of WPU1/P(PEGMA-co-GMAy) copolymers, after the addition of droplets of water on the cut scratches. The optical microscope images of the healed samples were taken after full evaporation of the added water. As shown in Figure 11a, the scratch on the pure WPU1 film did not heal after addition of water on it. In contrast, all WPU1/P(PEGMA-co-GMAy) composites studied (Figure 11b–d), exhibited high healing ability. In fact, it can be clearly seen that after addition of water, the cracks have almost disappeared. 

Τhe mechanism of healing herein is based on the water-assisted motion of the copolymers in the WPU1 matrix. Since the P(PEGMA-co-GMA50) and P(PEGMA-co-GMA70) copolymers are water-soluble, the addition of water probably leads to the softening of the polymer chains in the composites, favoring interdiffusion or even interpenetration of the polymer chains. Next, the chain’s mobility brings the cracked surfaces closer, enabling possible healing routes like hydrogen bonding of the urethane groups in WPU1, hydrolysis of the epoxide groups of GMA by water as well as reaction of the epoxide groups with groups like –NH_2_, –OH or –COOH, that could be present in the polyurethane. In fact, GMA-based healing materials, originating from the reaction of epoxide group with amines, are reported in literature. In such systems, either copolymers of GMA comprise a matrix [68] or GMA is encapsulated as a healing agent in microcapsules or hollow fibers, embedded in a matrix [35,69]. In these cases, healing is apparently not reversible, as the GMA epoxy groups will react once in the scratched area. However, the amount of the GMA content is designed to be sufficient so as not to be depleted at once. Moreover, from initial studies on the self-organization of the P(PEGMA-co-GMAy) copolymers in aqueous media, we have found that these copolymers form spherical, micellar-type self-assemblies, with a hydrophobic GMA core, surrounded by a hydrophilic PEGMA shell. Thus, GMA moieties are protected in the hydrophobic cores of such self-organized structures. So, GMA will be exposed, and its epoxide groups will be able to react with groups that could be present in the polyurethane matrix, only when a scratch or cut happens, that might disintegrate the micelle. 

The WPU1/P(BA-co-GMA) composites, on the contrary, did not display analogous behavior. In this case, none or only marginal healing was observed after the addition of water on the scratch (Figure S6).

**Figure 12:**
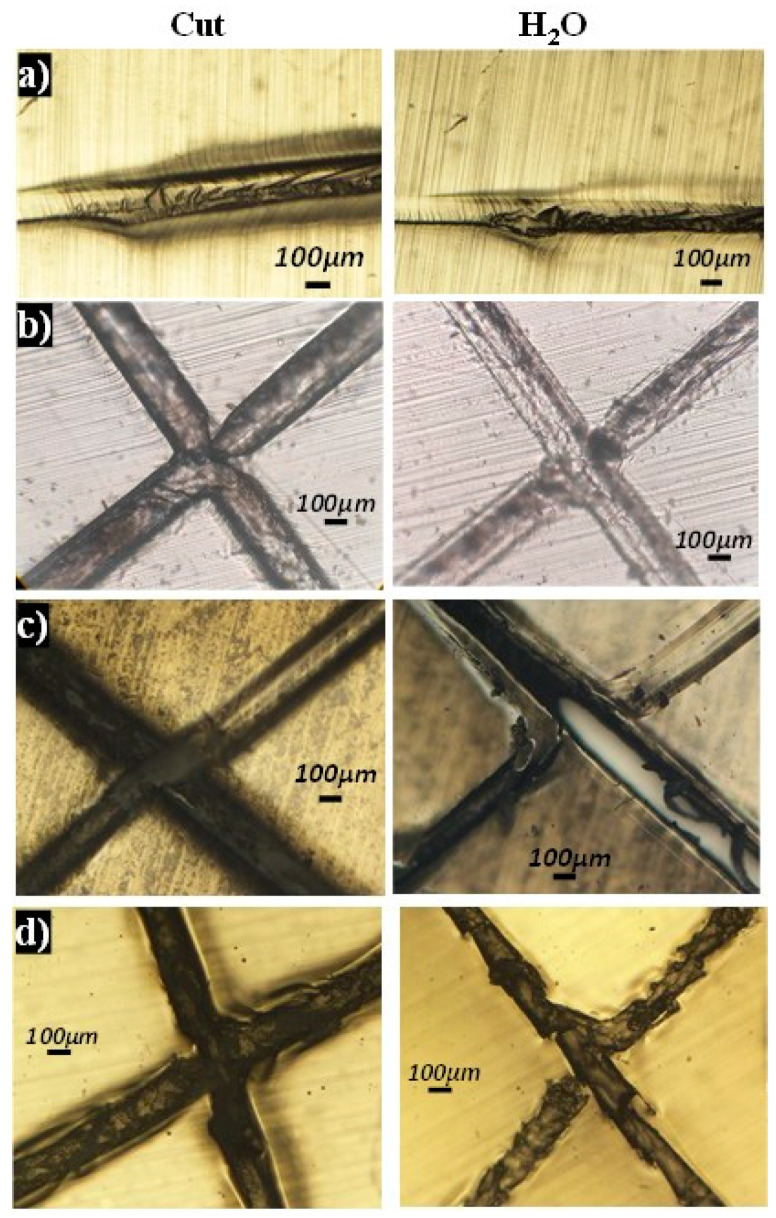
Optical microscopy images of cross scratches on (**a**) neat WPU2, (**b**) WPU2/10P(PEGMA-co-GMA50), (**c**) WPU2/20P(PEGMA-co-GMA50) and (**d**) WPU2/10P(PEGMA-co-GMA70) films, before (left photos) and after (right photos) addition of water on the cut.

Unlike the successful water-mediated healing performance of the WPU1/P(PEGMA-co-GMA) composites, the corresponding WPU2/xP(PEGMA-co-GMAy) composites did not show any healing tendency (Figure 12b–d), apart from the WPU2/10P(PEGMA-co-GMA70) film (Figure 12d), which showed a slight decrease of the cross-scratch width after the addition of water on the scratch. As already discussed in Table 4, the Tg values of the WPU2/10P(PEGMA-co-GMA50) and WPU2/20P(PEGMA-co-GMA50) composite films, indicated good compatibility of WPU2 and P(PEGMA-co-GMA50). This means that the P(PEGMA-co-GMA50) copolymer is well distributed in the WPU2 matrix. This compatibility, combined with the fact that WPU2 is a harder resin than WPU1 (Tg = −9 °C, Figure 8), suggests that the P(PEGMA-co-GMA50) copolymer is “immobilized” in the WPU2 matrix, not being able thus to move upon addition of water towards the scratch and promote healing, as in the case of the WPU1/P(PEGMA-co-GMAy) blends shown in Figure 11. Again here, it can be assumed that the governing behavior is based on the nature and the characteristics of the WPUs. 

**Figure 13:**
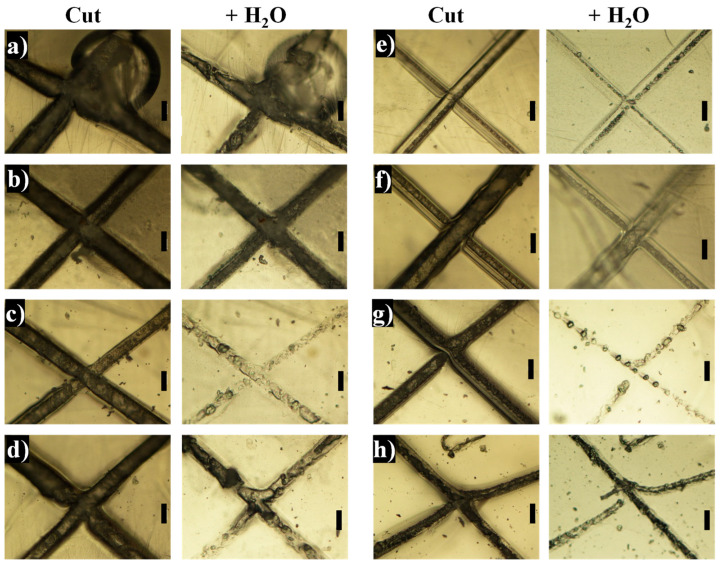
Optical microscopy images of scratches on (**a**) WPU2/10P(BA-co-GMA30), (**b**) WPU2/20P(BA-co-GMA30), (**c**) WPU2/10P(BA-co-GMA40), (**d**) WPU2/20P(BA-co-GMA40), (**e**) WPU2/10P(BA-co-GMA50), (**f**) WPU2/20P(BA-co-GMA50), (**g**) WPU2/10P(BA-co-GMA70) and (**h**) WPU2/20P(BA-co-GMA70) films, before (left photos) and after (right photos) addition of water on the cut. Scale bar is 100 μm.

The results of scratch heal recovery in the presence of water as a healing trigger for the WPU2/P(BA-co-GMA) blends are shown in Figure 13. It seems like the healing progress is enhanced as the molar ratio of GMA moieties is increased. For example, in Figure 13a,b, the WPU2/10P(BA-co-GMA30) film containing 10 and 20% *w*/*w* P(BA-co-GMA30) exhibit insignificant cure of the cut. As the GMA content increases in the respective films (Figure 13c–h), the scratches become smoother with a tendency to totally repair in some cases. In a recent study, Davydovich and Urban reported water accelerated self-healing in alternating/random hydrophobic acrylic-based copolymers (poly(methyl methacrylate/n-butyl acrylate) [p(MMA/nBA)] copolymers) [37]. In this study it was shown that the presence of confined water molecules in the proximity of ester groups may disrupt van der Waals interactions and participate in self-H-bonding. The unfavorable polymer-water interactions within hydrophobic domains will lead to the expulsion of water from the system. Then, polymer–polymer interactions due to enhanced interchain cohesive energies are generated. These interactions lead probably to potential fast repair. This is probably the case in our system too, were the P(BA-co-GMA) random, hydrophobic copolymer are used. What is more, in the present study, the healing process may not be only due to the water accelerated healing based on the interactions of BA, but also due to the presence of the reactive GMA moieties that will possibly proceed to reactions of the epoxide groups in the presence of water molecules, enhancing thus the healing progress. 

Though the healing process was not followed as a function of time, it is worthy to note that the aforementioned successful results have been observed 15 h after triggering. This healing time scale can be considered reasonable, taking into account that this is the upper time limit, while it can be further optimized for the intended practical application.

## 3. Materials and Methods

### 3.1. Materials

The monomers GMA (≥97%) and BA (≥99%), the macromonomer PEGMA (average molecular weight Mn = 950 g/mol) and the initiator azobisisobutyronitrile (AIBN) were purchased from Aldrich (Taufkirchen, Germany) and used as received. The solvents tetrahydrofuran (THF, anhydrous, p.a, ≥99.9%), 1,4-dioxane (p.a, ≥99.8%), hexane and diethyl ether (p.a, ≥99.5%) were obtained from Carlo Erba (Milano, Italy), while the solvent chloroform (p.a, ≥99.8%) was obtained from Honeywell (Ile de France, France). These solvents were used as received without further purification. Deuterated chloroform (CDCl_3_, 99.8% D) was purchased from Eurisotop (Saint-Aubin, France). The waterborne polyurethane dispersions WPU1 and WPU2 were kindly provided by Megara Resins SA (Megara, Greece). These WPUs have been synthesized through the prepolymer method, using the same diisocyanate agent. In WPU1, a polycarbonate polyol was utilized while in WPU2, a polyether-ester polyol was used. Moreover, it should be noted that WPU2 was a hybrid-alkyd PU, instead of WPU1 which was pure PU. 

Ultra-pure water was obtained by means of an Arium mini water purification system from Sartorius (Goettingen, Germany).

### 3.2. Synthesis of Copolymers

#### 3.2.1. Synthesis of P(BA-co-GMAy) Copolymers

The copolymers poly(n-butyl acrylate-co-glycidyl methacrylate) were synthesized via free radical copolymerization of BA and GMA monomers using AIBN as the initiator in THF at 70 °C. A typical polymerization reaction is as follows (e.g., P(BA-co-GMA70)) [70]. A round bottom flask, equipped with a magnetic stirrer and a reflux condenser, was charged under argon atmosphere with 10 mL BA (0.0698 mol), 21.5 mL GMA (0.1618 mol) and 108 mL THF, in order to acquire a concentration of ~30% *w*/*v*. The initiator AIBN was then added 0.305 g (1.9 mmol, 0.8 mol% over the total monomer concentration). The solution was degassed and left under inert atmosphere, while vigorously stirred in an oil bath at 70 °C for 1 day. The product was received by precipitation in hexane, filtered, washed with hexane and dried in a vacuum oven at 40 °C for 24 h. The actual mole fraction of GMA units in the copolymer P(BA-co-GMAy) was determined through Proton Nuclear Magnetic Resonance (^1^H-NMR) characterization in CDCl_3_. Moreover, the molecular weight of the copolymers was determined through size exclusion chromatography (SEC). in chloroform. In total, four copolymers P(BA-co-GMAy) were synthesized with feed composition y = 30, 40, 50, 70% moles GMA. A PBA and a PGMA homopolymer were synthesized for reasons of comparison. 

#### 3.2.2. Synthesis of P(PEGMA-co-GMAy) Copolymers

Four P(PEGMA-co-GMAy) copolymers (with feed compostion y = 40, 50, 60 or 70% moles in GMA) were synthesized through conventional radical polymerization of the macromonomer PEGMA and the monomer GMA, in presence of AIBN as initiator. In a three neck round bottom flask, PEGMA and GMA were dissolved in dioxane. The total monomers’ concentration in the organic solvent was 0.7 M. The mixture was degassed with nitrogen bubbling for ~30 min, followed by the addition of the initiator AIBN (1 mol% over the total monomer concentration). The final mixture was degassed with nitrogen for 1 h and the reaction temperature was set at 80 °C. Polymerization was carried out for 24 h. The copolymers with 40 and 50 %mol GMA content in feed (P(PEGMA-co-GMA40) and P(PEGMA-co-GMA50)) were recovered by adding in the copolymer mixture equal to dioxane volume of ultra-pure water. The final products were purified from monomers, impurities, etc., through dialysis against ultra-pure water (using a dialysis membrane with a MWCO of 12,000–14,000 Da) and were received in solid state through freeze drying. On the contrary, for the copolymers with 60 and 70 %mol GMA content in feed (P(PEGMA-co-GMA60) and P(PEGMA-co-GMA70)), the reaction mixture was precipitated in excess volume of diethyl ether. The precipitate was filtered, subsequently washed with diethyl ether and finally dried in a vacuum oven at 40 °C. The actual mol fractions of PEGMA and GMA were determined by ^1^H-NMR, using CDCl_3_ as solvent. The number and weight average molecular weight of the copolymers were determined by SEC. For comparison reasons, a P(PEGMA) homopolymer was similarly synthesized.

### 3.3. Preparation of Polyurethane/Polymer Films

For the preparation of the films from blends of polyurethane dispersions (WPU) and GMA copolymers, the following experimental procedure was followed: initially, aqueous solutions of P(PEGMA-co-GMAx) copolymers or solutions of P(BA-co-GMAy) in THF were prepared, dissolving a sufficient mass of each copolymer in a suitable volume of ultra-pure water or THF, respectively (the concentration of polymers was in the order of 6–10 %wt). The solutions were stirred at room temperature until full dissolution. Next, considering the solid content in PU of the WPU dispersions, appropriate amounts of the copolymers’ solutions were mixed with the WPU dispersions to achieve the desired copolymer mass content on dry basis, x (% *w*/*w*). Films with x = 10% *w*/*w* and 20% *w*/*w* were prepared. The mixtures were gently stirred to become homogenous and then poured into petri dishes or on Teflon sheet and allowed to stand for about ten days until the solvent was completely evaporated. The films had a thickness of ~0.3 mm. In addition, pure WPU films without GMA-based copolymers as additives were also prepared as reference. 

### 3.4. Characterization Techniques

#### 3.4.1. Proton Nuclear Magnetic Resonance (^1^H-NMR)

^1^H-NMR spectra of P(BA-co-GMAy) and P(PEGMA-co-GMAy) copolymers in CDCl_3_ were obtained at 400 MHz and at 25 °C using a Bruker AVANCE DPX 400 spectrometer (Billerica, MA, USA).

#### 3.4.2. Size Exclusion Chromatography (SEC)

The number average molecular weight (Mn) and weight average molecular weight (*M*_w_) as well as the polydispersity (PDI) of the P(BA-co-GMAy) and P(PEGMA-co-GMAy) copolymers were determined by SEC at 25 °C using a Marathon II HPLC (Rigas Labs, Thessaloniki, Greece) instrument with chloroform as the mobile phase and equipped with a Fasma 500 UV/vis detector, and two PLgel 5 μm Mixed columns (Agilent Technologies, SCC, Santa Clara, CA, USA). The flow rate of chloroform was set at 1 mL/min, while linear polystyrene standards were used for calibration of the columns. The software Clarity v.3.0.07.662 (DataApex Ltd., Prague, Czech Republic) was used for the spectra analysis. 

#### 3.4.3. Attenuated Total Reflection Fourier Transform Infrared Spectroscopy (ATR-FTIR) 

The ATR-FTIR spectra of the copolymers and PUD/copolymer films were recorded on a Bruker Platinum ATR-FTIR spectrometer (Billerica, MA, USA).

#### 3.4.4. Differential Scanning Calorimetry (DSC)

The thermal processes of pure polymers, pure WPUs films and WPU/polymer composite films were studied by DSC using a TA Instruments Q100 thermal analyzer (New Castle, DE, USA). All measurements were performed in a nitrogen atmosphere (50 mL/min). The specimens were heated from −100 to 100 °C at a heating rate of 10 °C/min and subsequently cooled to −100 °C at a cooling rate of 10 °C/min. Thermal transitions were obtained from first run, since the information from the first heating cycle refers to the actual state of the polymer. 

#### 3.4.5. Self-Healing Tests

For the self-healing study, the polyurethane/polymer films were scratched with a razor blade. Then, the films were either heated to 80 °C for 15 h or droplets of water were added on the scratch by a micropipette and left to dry at room temperature. The healing process of the films was monitored by a Nikon Eclipse L150 optical microscope (& Nikon’s NIS-Elements DS-U3 software) (Nikon metrology, Paris, France).

## 4. Conclusions

Two functional copolymer series, namely hydrophobic P(BA-co-GMAy) copolymers of GMA with n-butyl acrylate (BA) and amphiphilic copolymers P(PEGMA-co-GMAy) of GMA with a poly(ethylene glycol) methyl ether methacrylate (PEGMA) macromonomer, were designed and evaluated as potential healing agents of waterborne polyurethanes (WPUs) films. Homogenous composite films are obtained through the solution casting of WPU/P(BA-co-GMAy) or WPU/P(PEGMA-co-GMAy) mixtures for the studied compositions (the copolymer content is up to 20% *w*/*w*). The hybrid WPU/P(BA-co-GMAy) composites are amorphous, while the WPU/P(PEGMA-co-GMAy) vary from amorphous to semi-crystalline, depending on copolymer or blend composition. 

Two potential external healing triggers, namely heating at 80 °C or the addition of water, were explored. From the overall study, it is evidenced that the nature of the WPU used is decisive for the observation of healing behavior. In fact, the heating-triggered healing efficiency of both types of copolymers was proved limited. In contrast, these copolymers have been proved very effective materials for the water-triggered healing of WPU films.

In the present work, we focus on the design of the copolymers, while initial scratch studies were explored to follow healing. Detailed studies are under way to fully comprehend the behavior and to optimize the systems in terms of copolymers and blending composition. In the designed studies, mechanical tests are of utmost importance, to evaluate the influence of blending on the mechanical properties of WPUs, as well as the mechanical recovery after healing. Nevertheless, these initial studies evidence the potential of the novel materials as healing agents of WPUs or alternate polymer coatings upon water- or temperature- triggering. Moreover, the use of traditional free radical copolymerization techniques for the synthesis of such copolymers allows the design and facile development of additional functional copolymer families with potentially improved healing abilities, taking advantage of non-covalent interactions (hydrogen bonding, van der Waals, ionic interactions) through the selection and copolymerization of adequate monomers.

## Figures and Tables

**Table 1:** Characterization results for the P(BA-co-GMAy) copolymers.

Polymers	Feed Composition (% w GMA)	Feed Composition (% mol GMA)	^1^H-NMR Composition (% mol GMA)	*M*_n_ (g/mol)	*M*_w_ (g/mol)	PDI
P(BA-co-GMA30)	32	30	30	14,000	28,000	2.0
P(BA-co-GMA40)	42	40	46	23,000	55,000	2.4
P(BA-co-GMA50)	53	50	55	24,000	52,000	2.2
P(BA-co-GMA70)	72	70	67	21,000	45,000	2.1

**Table 2:** Characterization results for the P(PEGMA-co-GMAy) copolymers.

Polymers	Feed Composition (% w GMA)	Feed Composition (% mol GMA)	^1^H-NMR Composition (% mol GMA)	*M*_n_ (g/mol)	*M*_w_ (g/mol)	PDI (*M*_w_/*M*_n_)
P(PEGMA-co-GMA40)	9	40	45	60,000	105,000	1.7
P(PEGMA-co-GMA50)	13	50	54	235,000	470,000	2.0
P(PEGMA-co-GMA60)	18	60	67	80,000	220,000	2.7
P(PEGMA-co-GMA70)	26	70	74	110,000	340,000	3.1

**Table 3:** DSC results for the P(PEGMA-*co*-GMAy) copolymers.

Polymers	Tg (°C)	Tc (°C)	ΔHc (J/g)	Tm (°C)	ΔHm (J/g)	%Xc	%X’c
P(PEGMA)	−70	-	-	42	120.0	61.0	61.0
PGMA	70	-	-	-	-	-	-
P(PEGMA-co-GMA40)	−43	-	-	37	84.0	42.6	47.3
P(PEGMA-co-GMA50)	−45	-	-	31	99.2	50.3	59.2
P(PEGMA-co-GMA60)	−55	−24	39.6	25	77.7	19.3	25.1
P(PEGMA-co-GMA70)	−50	−23	33.8	31	73.2	20.0	28.6

**Table 4:** Characteristic glass and melting transition temperatures of neat polyurethane films, P(PEGMA-co-GMA) and P(BA-co-GMA) copolymers and the combination of both polyurethanes and copolymer in 90/10 *w*/*w* or 80/20 *w*/*w*.

Sample	Tg (°C)	Tc (°C)	ΔHc (J/g)	Tm (°C)	ΔHm (J/g)	%X″c
Pure WPU films						
WPU1	−23	-	-	-	-	-
WPU2	−9	-	-	-	-	-
Copolymers						
P(PEGMA-co-GMA50)	−45	-	-	31	99.2	59.2
P(PEGMA-co-GMA70)	−50	−23	33.80	31	73.20	28.6
WPU/copolymer films						
WPU1/ 10 P(PEGMA-co-GMA50)	−37	-	-	28	6.04	36.0
WPU1/ 20 P(PEGMA-co-GMA50)	−34	-	-	30.5	10.16	30.4
WPU1/ 10 P(PEGMA-co-GMA70) *	−32	-	-	-	-	-
WPU2/ 10 P(PEGMA-co-GMA50)	−28	-	-	-	-	-
WPU2/ 20 P(PEGMA-co-GMA50)	−32	-	-	26.5	6.50	19.4
WPU2/ 10 P(PEGMA-co-GMA70) *	−3	-	-	-	-	-

* DSC thermograms are shown in Figure S4.

## Data Availability

The data presented in this study are available on request from the corresponding author.

## Supplementary Materials

The following supporting information can be downloaded at: https://www.mdpi.com/article/10.3390/ijms23158118/s1.
