# Supplementary material for: Glycidyl Methacrylate-Based Copolymers as Healing Agents of Waterborne Polyurethanes
Source: Int J Mol Sci. 2022 Jul 23;23(15):8118. doi: 10.3390/ijms23158118 (PMC9332020; doi:10.3390/ijms23158118)
Supplement: Supplementary file 1 [file ijms-23-08118-s001.zip › ijms-1818653-supplementary.pdf]

Supporting Information

Glycidyl Methacrylate-based Copolymers as Healing Agents of Waterborne Polyurethanes

Ioanna Tzoumani [1], Amaia Soto Beobide [1,2], Zacharoula Iatridi [1,*], George A. Voyiatzis [2], Georgios Bokias [1], Joannis K. Kallitsis [1]

[1] Department of Chemistry, University of Patras, GR-26504, Patras, Greece; tzoumani@upnet.gr (I.T.); asoto@iceht.forth.gr (A.S.B.); iatridi@upatras.gr (Z.I); bokias@upatras.gr (G.B); kallitsi@upatras.gr (J.K.K)
[2] FORTH/ICE-HT, Stadiou Street, P.O. Box 1414, GR-26504, Patras, Greece; asoto@iceht.forth.gr (A.S.B.); gvog@iceht.forth.gr (G.A.V.)
*Correspondence: Zacharoula Iatridi, Department of Chemistry, University of Patras, GR-26504, Patras, Greece; E-mail address: iatridi@upatras.gr

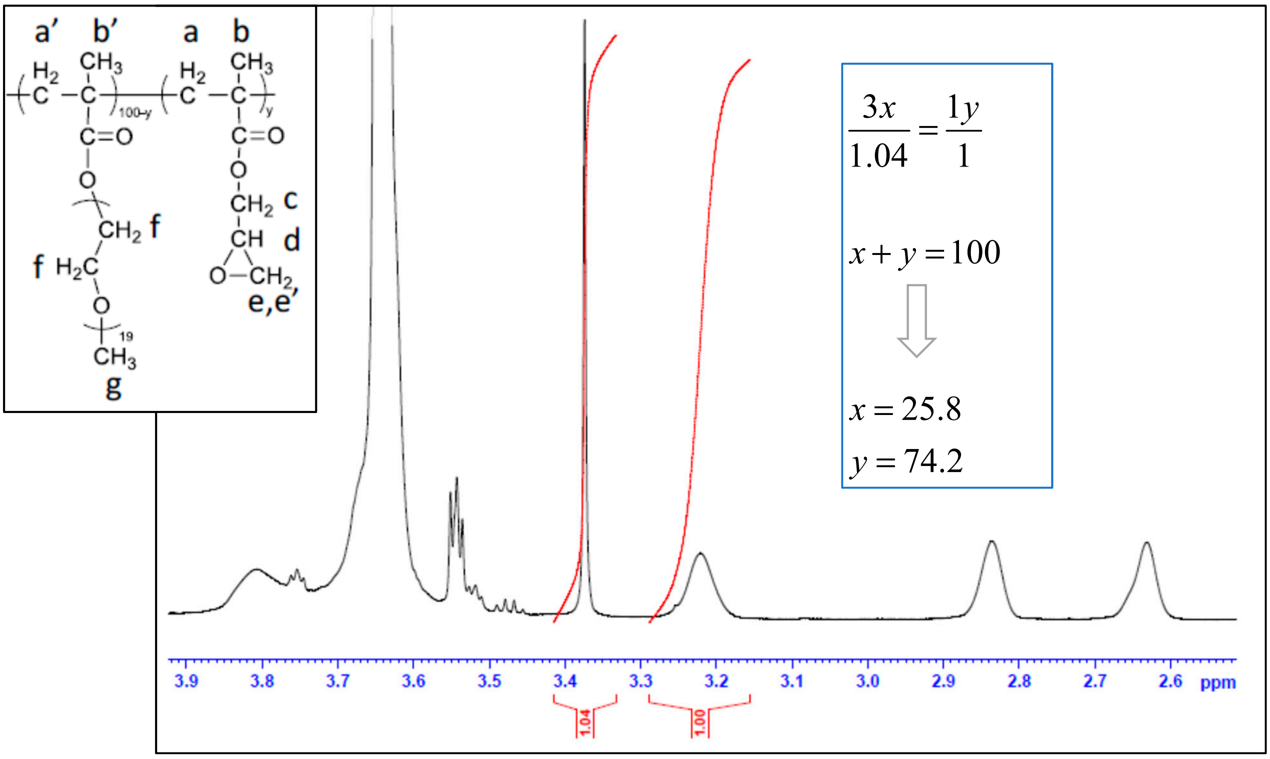

Figure S1. Zoom of the peaks at 3.26 and 3.38 ppm and their integral numbers from the ^{1}H-NMR spectrum of P(PEGMA-co-GMA70) copolymer.

$$\frac{3x}{1.04} = \frac{1y}{1} \tag{1}$$

$$x + y = 100 \tag{2}$$

In **Figure S1**, a zoom-in and integral numbers of the peaks at 3.26 and 3.38 ppm in the ^{1}H-NMR spectrum of P(PEGMA-co-GMA70) copolymer, is presented. The peak at 3.26 ppm corresponds to the proton of the -CH- group (d) of GMA, while the peak at 3.38 ppm is attributed to the 3 protons of the methylene group $-CH_3$ of PEGMA (g). Using the equations 1 and 2 the % molecular composition of the macromonomer PEGMA (x or 100-y) and the monomer GMA (y) can be calculated. Eq.1 is basically the ratio of the number of protons at 3.38 and 3.26 ppm over their integrals, while eq.2 shows that the sum of the numbers x and y is 100. The molecular composition of all P(PEGMA-co-GMAy) copolymers was calculated following the same procedure. The integrations of the peaks at 3.26 and 3.38 ppm for all P(PEGMA-co-GMAy) are shown in **Table S1**.

As far as the P(BA-co-GMAy) copolymers are concerned, a similar procedure was used. In this case, the integrals of the signals at the peaks 3.26 ppm (corresponds to the proton of the -CH- group (d) of GMA) and 1.39 ppm (methylene protons $-CH_2-$ (i) of BA) were used for the molecular characterization. The integrations of the peaks at 3.26 and 1.39 ppm for all P(BA-co-GMAy) are shown in **Table S2**. The equations used here are Eq.3 and Eq.4:

$$\frac{2x}{integral\ at\ 1.39\ ppm} = \frac{1y}{\text{integral at 3.26 ppm}} \tag{3}$$

$$x + y = 100 \tag{4}$$

Table S1. The integrals of the peaks at 3.26 and 3.38 ppm for the P(PEGMA-co-GMAy) copolymers.

Polymers	Feed composition (% mol GMA)	Integration of peak at 3.38 ppm / number of protons	Integration of peak at 3.26 ppm / number of protons	^{1}H-NMR composition (% mol GMA)
**P(PEGMA-co-GMA40)**	40	3.68 / 3	1 / 1	45
**P(PEGMA-co-GMA50**)	50	2.59 / 3	1 / 1	54
**P(PEGMA-co-GMA60)**	60	1.46 / 3	1 / 1	67
**P(PEGMA-co-GMA70)**	70	1.04 / 3	1 / 1	74

Table S2. The integrals of the peaks at 3.26 and 1.39 ppm for the P(BA-co-GMAy) copolymers.

Polymers	Feed composition (% mol GMA)	Integration of peak at 1.39 ppm / number of protons	Integration of peak at 3.26 ppm / number of protons	^{1}H-NMR composition (% mol GMA)
**P(BA-co-GMA30)**	30	4.4111 / 2	0.9434 / 1	30
**P(BA-co-GMA40)**	40	2.3444 / 2	0.9939 / 1	46
**P(BA-co-GMA50)**	50	1.6244 / 2	1.0063 / 1	55
**P(BA-co-GMA70)**	70	0.9479 / 2	0.9833 / 1	67

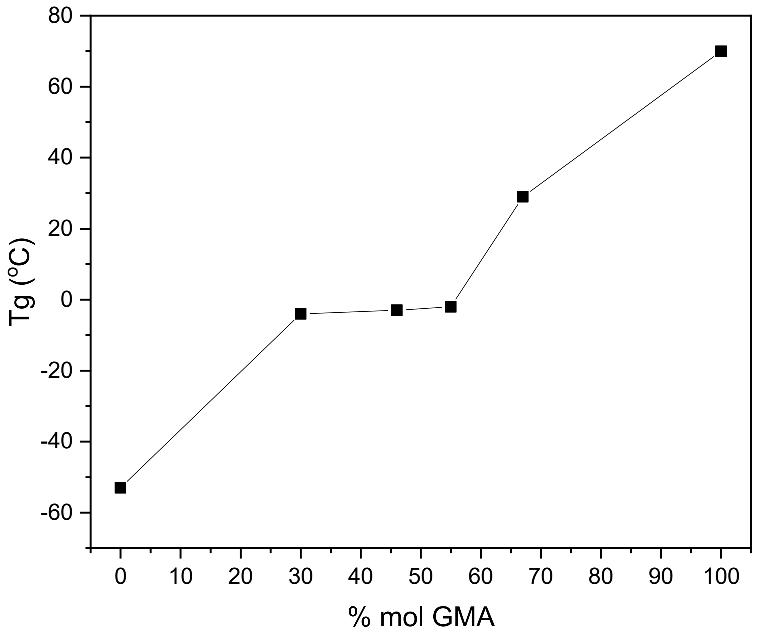

Figure S2. Plot of Tg versus the % mol GMA content of the P(BA-co-GMAy) copolymers, obtained from DSC.

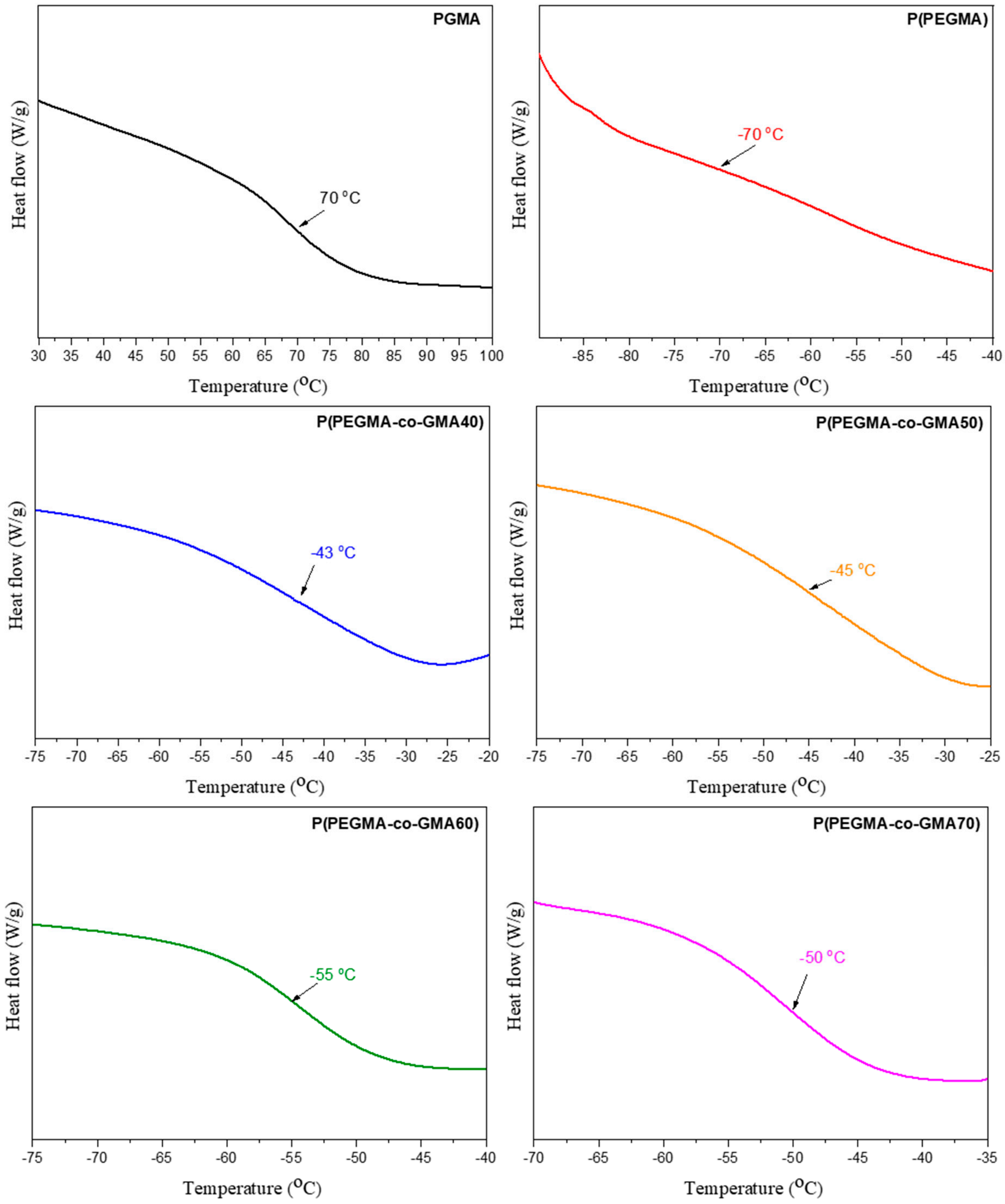

Figure S3. Zoomed areas of the DSC curves of first heating cycle for the P(PEGMA-co-GMAy) copolymers and the P(PEGMA) and PGMA homopolymers to observe the Tg.

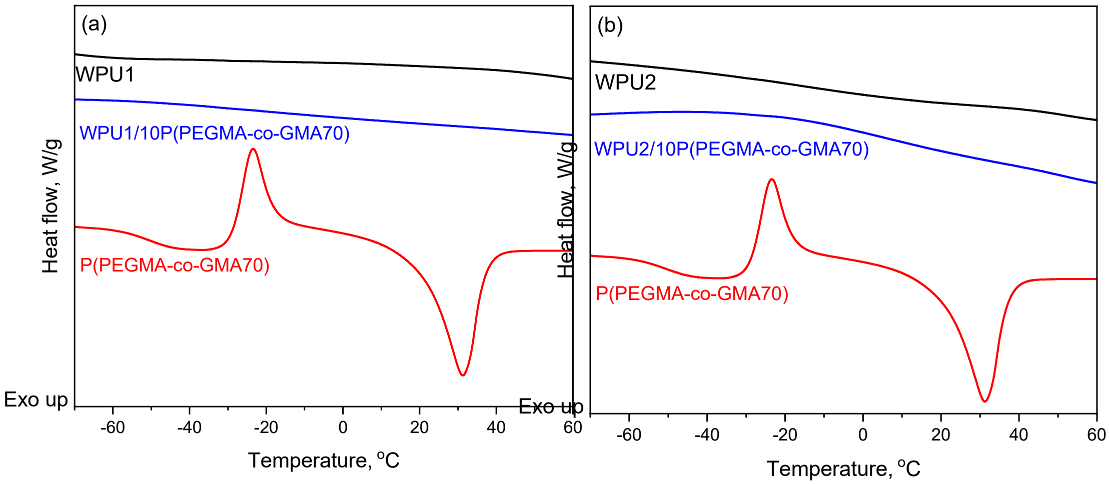

Figure S4. DSC of the a) WPU1/P(PEGMA-co-GMA70) and b) WPU2/P(PEGMA-co-GMA70) films containing P(PEGMA-co-GMA70) (10 %wt). P(PEGMA-co-GMA70) copolymer and the films of pure WPU1 and WPU2 are also presented.

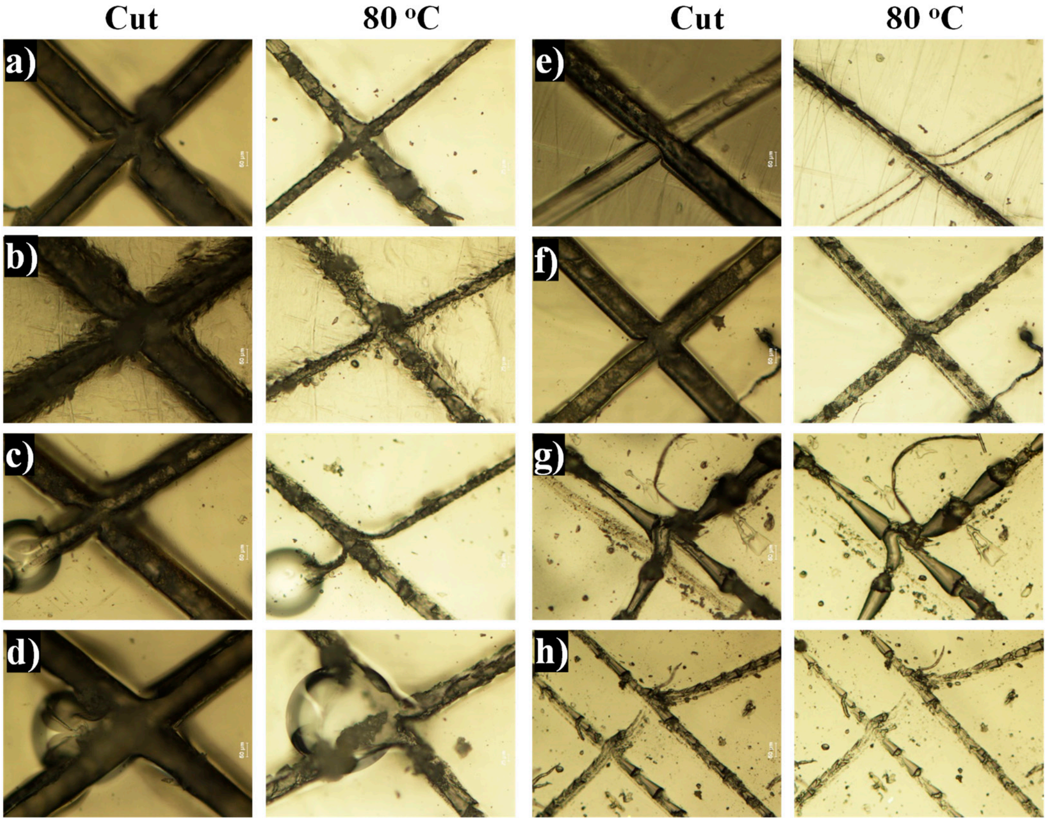

Figure S5. Optical microscopy images of scratches on (a) WPU2/10P(PBA-co-GMA30), (b) WPU2/20P(PBA-co-GMA30), (c) WPU2/10P(PBA-co-GMA40), (d) WPU2/20P(PBA-co-GMA40), (e) WPU2/10P(PBA-co-GMA50), (f) WPU2/20P(BA-co-GMA50), (g) WPU2/10P(BA-co-GMA70) and (h) WPU2/20P(BA-co-GMA70) films, before (left photos) and after (right photos) heating the films at 80 °C.

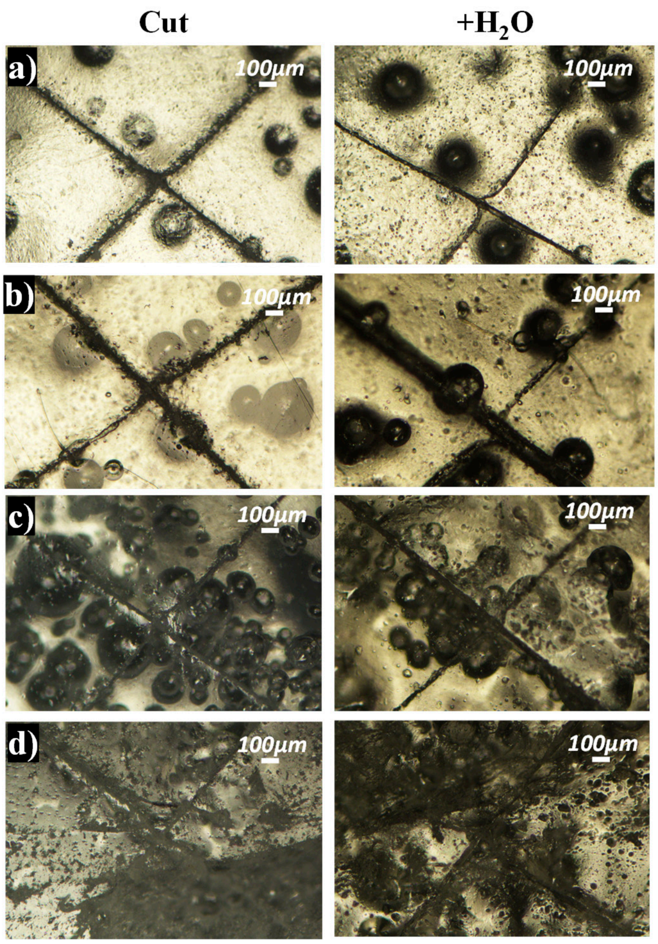

Figure S6. Optical microscopy images of cross scratch on (a) WPU1/10P(BA-co-GMA30), (b) WPU1/10P(BA-co-GMA40), (c) WPU1/10P(BA-co-GMA50) and (d) WPU1/10P(BA-co-GMA70) films, before (left photos) and after (right photos) addition of water on the cut.
